# Food Predictors of Plasma Carotenoids

**DOI:** 10.3390/nu5104051

**Published:** 2013-10-11

**Authors:** Sara J. Hendrickson, Walter C. Willett, Bernard A. Rosner, A. Heather Eliassen

**Affiliations:** 1 Department of Epidemiology, Harvard School of Public Health, Boston, MA 02115, USA; E-Mails: nhsjh@channing.harvard.edu (S.J.H.); wwillett@hsph.harvard.edu (W.C.W.); 2 Department of Nutrition, Harvard School of Public Health, Boston, MA 02115, USA; 3 Channing Division of Network Medicine, Department of Medicine, Brigham and Women’s Hospital and Harvard Medical School, Boston, MA 02115, USA; E-Mail: stbar@channing.harvard.edu

**Keywords:** carotenoids, vitamin A, α-carotene, β-carotene, β-cryptoxanthin, lutein/zeaxanthin, lycopene, food predictors, biomarkers

## Abstract

Empirical prediction models that weight food frequency questionnaire (FFQ) food items by their relation to nutrient biomarker concentrations may estimate nutrient exposure better than nutrient intakes derived from food composition databases. Carotenoids may especially benefit because contributing foods vary in bioavailability and assessment validity. Our objective was to develop empirical prediction models for the major plasma carotenoids and total carotenoids and evaluate their validity compared with dietary intakes calculated from standard food composition tables. 4180 nonsmoking women in the Nurses’ Health Study (NHS) blood subcohort with previously measured plasma carotenoids were randomly divided into training (*n* = 2787) and testing (*n* = 1393) subsets. Empirical prediction models were developed in the training subset by stepwise selection from foods contributing ≥0.5% to intake of the relevant carotenoid. Spearman correlations between predicted and measured plasma concentrations were compared to Spearman correlations between dietary intake and measured plasma concentrations for each carotenoid. Three to 12 foods were selected for the α-carotene, β-carotene, β-cryptoxanthin, lutein/zeaxanthin, lycopene, and total carotenoids prediction models. In the testing subset, Spearman correlations with measured plasma concentrations for the calculated dietary intakes and predicted plasma concentrations, respectively, were 0.31 and 0.37 for α-carotene, 0.29 and 0.31 for β-carotene, 0.36 and 0.41 for β-cryptoxanthin, 0.28 and 0.31 for lutein/zeaxanthin, 0.22 and 0.23 for lycopene, and 0.22 and 0.27 for total carotenoids. Empirical prediction models may modestly improve assessment of some carotenoids, particularly α-carotene and β-cryptoxanthin.

## 1. Introduction

Food frequency questionnaires (FFQ) are often used to assess usual dietary intake in epidemiologic studies. Responses are typically translated to nutrient intakes by multiplying reported consumption frequencies of commonly-used units or portion sizes of food items by the nutrient contents of the specified unit and summing over all foods. Nutrient contents are determined from several sources, including the USDA nutrient database [1], scientific journal articles, and manufacturer information. However, an individual’s nutrient intake determined this way may not adequately represent their internal dose, which is often more etiologically-relevant. This potential inadequacy may be due to factors such as responder interpretation of the FFQ; nutrient content data inaccuracy; individual differences in absorption, metabolism, or other physiologic or lifestyle factors; and nutrient bioavailability [2]. An alternative method is to utilize empirical prediction models that weight foods by their relation to biological nutrient (biomarker) levels, such as plasma concentrations [2]. This method reduces error from inaccurate food composition data, bioavailability differences among foods, and variation in the validity of questions on individual foods. Error may be introduced if the empirical weights are imprecise or not generalizable to the population of interest [2]. Whether error associated with empirical weight-based methods impacts validity more or less than error associated with nutrient composition-based methods is generally unknown. The impact may vary across studies because the precision of the empirical weights and thus degree of random error depends on sample size.

Empirical prediction models may be particularly suited for nutrients obtained from foods with highly varying bioavailability and assessment validity. One such class of nutrients is carotenoids, acquired mainly from fruit and vegetable intake. Carotenoid bioavailability varies across different foods and different forms of the same food [3,4,5,6]. Based on the correlation corrected for within-person variation, assessment validity with respect to diet records is good for some carotenoid-containing foods (e.g., orange/grapefruit juice (*r* = 0.84), eggs (*r* = 0.77)), but lower for others (carrots (*r* = 0.40), yellow squash (*r* = 0.49)) [7]. Given observed inverse associations between dietary intake of certain carotenoids and estrogen receptor negative breast cancer [8] and late age-related macular degeneration [9], improved carotenoid assessment is of interest. We developed empirical prediction models for plasma concentrations of individual and total carotenoids and compared the correlations with measured plasma concentrations for the predicted plasma concentrations versus calculated dietary intakes. We thus extend a previous β-carotene analysis [10] to other carotenoids, a larger sample size, and updated nutrient composition data.

## 2. Subjects and Methods

The Nurses’ Health Study (NHS) was established in 1976 when 121,700 female registered nurses in the United States aged 30–55 completed a mailed questionnaire regarding medical history and other health-related exposures. The women have been followed biennially by mailed self-administered questionnaires. In 1989–1990, 32,826 NHS participants provided blood samples. Details of the blood collection have been published previously [11]. In brief, women arranged to have their blood drawn and shipped, via overnight courier with an icepack, to our laboratory where samples were immediately centrifuged, aliquoted, and stored in liquid nitrogen freezers. Ninety-seven percent of samples were received within 26 h of collection.

This study includes participants from nested case-control studies of breast cancer (*n* = 2313), cataract (*n* = 652), colorectal cancer (*n* = 361), colorectal adenoma (*n* = 589), and myocardial infarction (*n* = 265) for whom plasma carotenoid concentrations were assayed. To avoid subclinical breast or colon cancer impacting carotenoid intake or bioavailability, women diagnosed with either disease ≤2 years after blood collection were excluded (*n* = 168).

Plasma carotenoid concentrations were assayed in batches by the Micronutrient Analysis Laboratory in the Department of Nutrition at the Harvard School of Public Health using reverse-phase HPLC by the methods described by El-Sohemy *et al.* [12]. Blinded quality control samples (10%) were randomly placed throughout batches and technicians were blinded to case-control status. Coefficients of variation (CV) were calculated within each laboratory batch. Across the 12 batches for each biomarker, CVs were generally <15% except for one batch for β-carotene (CV = 20.7%), two batches for β-cryptoxanthin (CVs = 19.4%, 20.6%), and one batch for lycopene (CV = 17.5%). Total carotenoids were summed from the five assayed carotenoids. One β-carotene, 4 β-cryptoxanthin, and thus 5 total carotenoids values were missing due to laboratory technical difficulties.

Total plasma cholesterol was assayed in 15 batches using the enzymatic methods described by Allain *et al.* [13]. Coefficients of variation were 2.1%–15.6%. Plasma cholesterol data were not available for 426 women.

All included participants completed FFQs in 1986 and 1990. FFQs were considered complete if a woman reported a plausible total energy intake (600–3500 kcal/day), left ≤70 food items blank, and did not skip the fruit or vegetable sections. Nutrient intakes were calculated by multiplying reported consumption frequencies of commonly-used units or portion sizes of food items by the nutrient contents of the specified unit and summing over all foods. Food nutrient values were derived primarily from USDA sources. Use of multivitamins and other supplements, as well as dose and duration of use, were incorporated into β-carotene intake. The FFQ reproducibility and validity has been reported previously [7,14,15]. The correlation between FFQ-estimated vitamin A intake from food and supplements *versus* 4, 1-week diet records was 0.49; validity for specific carotenoids was not calculated [15]. Carotenoid intakes were adjusted for total energy intake by the residual method [2]. As the association between dietary β-carotene intake and plasma β-carotene concentrations may be obscured by threshold effects among women with high supplemental β-carotene intake (e.g., dietary intake may impact plasma levels to a lesser extent much among women taking supplements), 107 women who reported β-carotene supplement use and/or had a supplemental β-carotene intake ≥5054 μg/day (the amount in one medium carrot [1]) in 1990 were excluded from the β-carotene and total carotenoids analyses.

To reduce within-person variation, nutrient and food intakes were averaged from the 1986 and 1990 FFQs, except for six items on the 1986 but not 1990 FFQ.

Non-dietary variables were obtained from biennial or blood collection questionnaires. Menopausal status and postmenopausal hormone use were determined from a questionnaire completed at the time of blood collection. Date of birth and height were determined from the baseline NHS questionnaire. Body mass index (BMI) was calculated using height reported in 1976 and weight reported on the blood collection questionnaire, or the 1990 (*n* = 104) or 1998 (*n* = 2) questionnaire if missing. The median BMI value for all women was used for women missing all weights or height (*n* = 5). Smoking status was determined from the 1990 NHS questionnaire; 659 women reporting current smoking were excluded. The final total sample included 4180 women, of whom 2241 were controls. The study protocol was approved by the Institutional Review Board of Brigham and Women’s Hospital.

### Statistical Analysis

Plasma carotenoid values were natural log-transformed to improve normality. Outlying carotenoid concentrations were identified within batch by a generalized extreme Studentized deviate many-outlier detection method [16]; outlying plasma cholesterol concentrations were identified across all batches by the same method. Consequently, 3–21 values of individual carotenoids and 2 cholesterol values were set to missing. To account for laboratory variation that was evident across batches due to batches being assayed at different times, plasma carotenoid concentrations were standardized to an average batch with a method used previously to account for study effects among eleven blood pressure studies [17].

To remove variation in plasma carotenoid levels due to non-dietary factors, we obtained residuals from multivariate linear regression of each natural log-transformed plasma carotenoid on the following covariates: age (years), case-control status (indicators for breast cancer, colorectal cancer, colorectal adenoma, myocardial infarction, and cataract), BMI, plasma cholesterol (mg/dL), and menopausal status and hormone therapy (HT) use (premenopausal; postmenopausal, no HT; postmenopausal, HT; unknown). For interpretability, the mean natural log-transformed plasma concentration of the relevant carotenoid was added back to each residual. These adjusted values are hereafter referred to as plasma concentrations and used in all analyses.

To select foods for the empirical prediction models and provide unbiased estimates of model performance after food selection, participants were randomly divided into two groups: a two-thirds training subset (*n* = 2787) and a one-third testing subset (*n* = 1393). Candidate food predictors were all foods contributing ≥0.5% to total intake of the relevant carotenoid in the full cohort (Table S1). Supplemental β-carotene (mg/day) in 1990 was also a candidate predictor for β-carotene and total carotenoids. Percent contribution of each food to total intake was determined by averaging the percentages across FFQs administered in 1986 and 1990. To determine each food’s average percent contribution to total carotenoids intake, each food’s average percent contribution to each carotenoid was weighted by each carotenoid’s percent contribution to total carotenoids intake in our sample (*i.e*., α-carotene = 5.4%, β-carotene = 28.8%, β-cryptoxanthin = 1.4%, lutein/zeaxanthin = 20.4%, and lycopene = 44.0%) and then summed across carotenoids for each food. In the training subset, foods were chosen by stepwise selection into linear regression models, using SAS PROC GLMSELECT, with the carotenoid concentration as the dependent variable and total energy intake forced into the model. Significance levels to enter and stay were 0.10 and 0.05, respectively.

Predicted plasma carotenoid concentrations in the testing subset were calculated from the regression models developed in the training subset. Spearman correlations were calculated between the predicted and measured plasma concentrations and between the calculated dietary intakes and measured plasma concentrations. To determine whether the correlations with measured plasma carotenoid concentrations were significantly different for the predicted plasma concentrations versus calculated dietary intakes, Wolfe’s Test for Comparing Dependent Correlation Coefficients [18] was performed after taking the probit transformation of each variable’s rank divided by (*n* + 1) in order to convert the ranks to a normally-distributed scale [19].

Correction of the Spearman correlation coefficients for random within-person variation was performed by first dividing the Pearson correlation coefficients for the probit[rank/(*n* + 1)]-transformed values [19] by the square root of the product of the intraclass correlations (ICCs) for the relevant measures of carotenoid status (*i.e*., measured plasma concentration and either calculated dietary intake or predicted plasma concentration). The corrected Pearson correlation coefficients were then converted back to Spearman correlation coefficients based on the relations presented in [19]. To capture medium-term variation, ICCs for natural log-transformed calculated dietary intakes were determined from the 1984 and 1986 FFQs for all NHS participants with available data. Similarly, ICCs for the natural log-transformed predicted plasma carotenoid concentrations were calculated among these women from food intakes reported on the 1984 and 1986 FFQs; outlying predicted plasma concentrations were removed [16]. Measured plasma carotenoid ICCs were previously reported for 40 NHS participants who provided two blood samples 1–2 years apart [20].

Interactions between selected foods and fat intake were assessed by including food-by-fat cross-product terms in the final selected models. Fat was quantified as grams per day, percent of energy per day, and salad dressing servings per day and modeled as each woman’s intake minus her data subset’s (*i.e*., training or testing) mean intake (residuals). Linear regression models including the relevant carotenoid’s selected foods, food-by-fat cross-products, and the main effect of fat were fit in the training subset. Separate models were run for each food-by-fat cross-product, and significant cross-products were included in the relevant model simultaneously. Final interaction models included only significant food-by-fat cross-products. These models were then applied to the testing subset to generate predicted plasma concentrations, which were compared to measured plasma concentrations and calculated dietary intakes as described above.

All reported *P* values are 2-sided and considered statistically significant at <0.05, and all statistical analyses were performed using SAS version 9 [21].

## 3. Results

Participant characteristics at blood collection are shown in Table 1. There were no appreciable differences between the training and testing subsets. Generally, the women were in their fifties or early sixties, normal-to-overweight, postmenopausal, and non-users of β-carotene supplements. Approximately 40% of the women used multivitamins.

**Table 1:** Participant characteristics at time of blood collection by dataset.

Characteristic	Training		Testing	
	*n*	Mean (SD ^1^) or %	*n*	Mean (SD) or %
Age (years)	2787	58.6 (6.9)	1393	58.5 (6.8)
Body mass index (kg/m^2^)	2787	25.6 (4.5)	1393	25.5 (4.6)
Multivitamin use	1116	40	578	41
β-carotene supplement use	72	3	30	2
Premenopausal	458	16	216	16
Postmenopausal, no HT ^1^	1077	39	567	41
Postmenopausal, HT	1013	36	500	36
Unknown menopausal status or HT	239	9	110	8
*Daily dietary intake (μg)* ^2^				
α-carotene	2787	776 (496)	1393	799 (509)
β-carotene	2787	4397 (2193)	1393	4442 (2208)
Supplemental β-carotene, 1990	2787	360 (1253)	1393	372 (1134)
β-cryptoxanthin	2787	183 (92)	1393	184 (94)
Lutein/zeaxanthin	2787	2955 (1635)	1393	2950 (1630)
Lycopene	2787	6336 (3219)	1393	6321 (3200)
Total carotenoids	2787	14,646 (5452)	1393	14,697 (5560)
*Plasma biomarker concentration* ^3^				
α-carotene	2784	74 (50)	1387	74 (52)
β-carotene	2706	291 (207)	1358	289 (212)
β-cryptoxanthin	2780	84 (46)	1390	83 (43)
Lutein/zeaxanthin	2786	187 (74)	1391	181 (68)
Lycopene	2775	425 (176)	1384	419 (177)
Total carotenoids	2702	1080 (403)	1356	1062 (394)
Plasma cholesterol (mg/dL)	2494	218 (39)	1258	217 (40)

^1^ Standard deviation (SD), hormone therapy (HT); ^2^ Energy-adjusted; ^3^ Plasma carotenoids (μg/L) adjusted for age, case-control status, body mass index, plasma cholesterol, menopausal status, and post-menopausal hormone use by the residual method.

Based on the model adjusted *R*^2^, the empirical prediction models explained 5% (lycopene) to 15% (α-carotene and β-cryptoxanthin) of the variation in plasma carotenoid concentrations in the training subset. The models explained 6% (lycopene) to 16% (β-cryptoxanthin) in the testing subset (Table 2, Table 3 and Table 4). The individual foods explaining the largest % variation (assessed by partial *R*^2^) in each carotenoid in the training subset were raw carrots for α-carotene, β-carotene, and total carotenoids; orange juice for β-cryptoxanthin; romaine or leaf lettuce for lutein/zeaxanthin; and tomato sauce for lycopene. Most of these foods also explained the largest % variation in the respective carotenoid in the testing subset (Table 2, Table 3 and Table 4); however, orange juice explained a slightly larger percent than raw carrots for total carotenoids.

**Table 2:** Plasma α-carotene, β-carotene, β-cryptoxanthin multivariate linear regression models ^1^.

Carotenoid	Food ^2^	Cohort	Training (*n* = 2706–2784)				Testing (*n* *=* 1358–1390)
		% ^3^	β	SE ^4^	*P*	Partial *R*^2^	Partial *R*^2^
α-carotene ^5,^^6^							
	Carrots, raw	29.5	0.704	0.036	<0.0001	0.121	0.107 ^7^
	Bananas	1.0	0.212	0.040	<0.0001	0.010	0.006 ^7^
	Carrots, cooked	45.9	0.418	0.087	<0.0001	0.008	0.009 ^7^
β-carotene ^8,9^							
	Carrots, raw	10.9	0.361	0.040	<0.0001	0.029	0.024 ^7^
	Supplemental β-carotene	6.9	0.130	0.0212	<0.0001	0.014	0.018 ^7^
	Broccoli	4.6	0.337	0.069	<0.0001	0.009	0.006 ^7^
	Lettuce, romaine or leaf	8.1	0.171	0.042	<0.0001	0.006	0.007 ^7^
	Cantaloupe	7.1	0.374	0.097	0.0001	0.005	0.007 ^7^
	Prunes	0.6	0.173	0.067	0.01	0.002	0.001
	Pizza	0.7	−0.466	0.191	0.01	0.002	0.001
β-cryptoxanthin ^10,11^							
	Juice, orange	38.3	0.267	0.019	<0.0001	0.066	0.080 ^7^
	Oranges	16.0	0.435	0.039	<0.0001	0.043	0.048 ^7^
	Peaches, apricots, or plums	6.3	0.233	0.048	<0.0001	0.008	0.012 ^7^
	Carrots, raw	3.4	0.136	0.031	<0.0001	0.007	0.001
	Apples or pears, fresh	2.4	0.124	0.029	<0.0001	0.007	0.007 ^7^
	Corn	6.6	−0.298	0.083	0.0003	0.005	0.000
	Prunes	3.1	0.130	0.053	0.01	0.002	0.003 ^7^
	Cucumbers	2.3	−0.064	0.028	0.02	0.002	0.001

^1^ Plasma concentrations were natural log transformed and adjusted for age, case-control status, body mass index, plasma cholesterol, menopausal status, and hormone therapy use by the residual method; ^2^ Foods (servings/day; milligrams/day for supplemental β-carotene) selected among the training subset by stepwise selection from all foods contributing ≥0.5% to specific carotenoid intake in the full cohort with 0.10 significance level to enter and 0.05 significance level to stay; ^3^ 1986–1990 average percent contribution to total intake in the full cohort; supplemental β-carotene intake is 1990 only; ^4^ Standard error (SE); ^5^ Intercept = 3.98, β (SE) for total energy intake = −0.0000798 (0.0000249); ^6^ Model adjusted *R*^2^ = 0.15 in training and 0.12 in testing; ^7^ *P* < 0.05; ^8^ Intercept = 5.36, β (SE) for total energy intake = −0.0000758 (0.0000274); ^9^ Model adjusted *R*^2^ = 0.09 in training and 0.08 in testing; ^10^ Intercept = 4.12, β (SE) for total energy intake = −0.0000602 (0.0000223); ^11^ Model adjusted *R*^2^ = 0.15 in training and 0.16 in testing.

**Table 3:** Plasma lutein/zeaxanthin, lycopene multivariate linear regression models ^1^.

Carotenoid	Food ^2^	Cohort	Training (*n* = 2775–2786)				Testing (*n* *=* 1384–1391)
		% ^3^	β	SE ^4^	*P*	Partial *R*^2^	Partial *R*^2^
lutein/zeaxanthin ^5,^^6^	Lettuce, romaine or leaf	7.8	0.164	0.026	<0.0001	0.014	0.025 ^7^
	Juice, orange	3.1	0.085	0.015	<0.0001	0.011	0.020 ^7^
	Broccoli	8.6	0.185	0.045	<0.0001	0.006	0.009 ^7^
	Spinach, cooked	21.7	0.334	0.105	0.002	0.004	0.001
	Carrots, raw	0.6	0.082	0.025	0.001	0.004	0.001
	Eggs	1.3	0.105	0.036	0.003	0.003	0.006 ^7^
	Spinach, raw	11.7	0.224	0.093	0.02	0.002	0.002
	Eggplant/zucchini/other summer squash	3.1	0.165	0.072	0.02	0.002	0.002
	Tomatoes	1.8	0.059	0.026	0.03	0.002	0.000
	Corn	2.5	−0.146	0.066	0.03	0.002	0.000
	Oranges	1.1	0.063	0.030	0.03	0.002	0.001
	Popcorn	0.6	0.045	0.022	0.04	0.002	0.007 ^7^
Lycopene ^8,9^							
	Tomato sauce	42.4	0.553	0.073	<0.0001	0.020	0.031 ^7^
	Pizza	15.2	0.648	0.137	<0.0001	0.008	0.007 ^7^
	Tomatoes	16.7	0.131	0.029	<0.0001	0.007	0.004 ^7^
	Juice, tomatoes	11.9	0.213	0.072	0.003	0.003	0.08 ^7^

^1^ Plasma concentrations were natural log transformed and adjusted for age, case-control status, body mass index, plasma cholesterol, menopausal status, and hormone therapy use by the residual method; ^2^ Foods (servings/day; milligrams/day for supplemental β-carotene) selected among the training subset by stepwise selection from all foods contributing ≥0.5% to specific carotenoid intake in the full cohort with 0.10 significance level to enter and 0.05 significance level to stay; ^3^ 1986–1990 average percent contribution to total intake in the full cohort; supplemental β-carotene intake is 1990 only; ^4^ Standard error (SE); ^5^ Intercept = 5.03, β (SE) for total energy intake = −0.0000612 (0.0000182); ^6^ Model adjusted *R*^2^ = 0.08 in training and 0.09 in testing; ^7^ *P* < 0.05; ^8^ Intercept = 5.91, β (SE) for total energy intake = −0.0000705 (0.0000198); ^9^ Model adjusted *R*^2^ = 0.05 in training and 0.06 in testing.

**Table 4:** Plasma total carotenoids multivariate linear regression model ^1,2,3^.

Food ^4^	Cohort	Training (*n* = 2702)				Testing (*n* *=* 1356)
	% ^5^	β	SE ^6^	P	Partial *R*^2^	Partial *R*^2^
Carrots, raw	4.9	0.176	0.024	<0.0001	0.019	0.017 ^7^
Lettuce, romaine or leaf	3.9	0.111	0.025	<0.0001	0.007	0.006 ^7^
Oranges	0.6	0.119	0.029	<0.0001	0.006	0.005 ^7^
Juice, orange	1.3	0.058	0.014	<0.0001	0.006	0.020 ^7^
Tomato sauce	19.4	0.242	0.061	<0.0001	0.006	0.012 ^7^
Broccoli	3.1	0.131	0.041	0.002	0.004	0.003
Corn	0.6	−0.188	0.063	0.003	0.003	0.000
Cantaloupe	2.1	0.126	0.059	0.03	0.002	0.003 ^7^
Tomatoes	9.4	0.054	0.025	0.03	0.002	0.000

^1^ Plasma total carotenoid concentrations were natural log transformed and adjusted for age, case-control status, body mass index, plasma cholesterol, menopausal status, and hormone therapy use by the residual method; ^2^ Intercept = 6.84, β (SE) for total energy intake = −0.0000649 (0.0000174); ^3^ Model adjusted *R*^2^ = 0.07 in training and 0.08 in testing; ^4^ Foods (servings/day) selected among the training subset by stepwise selection from all foods contributing ≥0.5% to total carotenoids intake in the full cohort with 0.10 significance level to enter and 0.05 significance level to stay; ^5^ 1986–1990 average percent contribution to total intake in the full cohort; supplemental β-carotene is 1990 only; ^6^ Standard error (SE); ^7^ *P* < 0.05.

The Spearman correlations with measured plasma concentrations were higher for predicted than calculated dietary intake for all carotenoids in both subsets, excluding lycopene in the training subset (Table 5). In the training subset, the correlations with measured plasma concentration were significantly different between calculated diet and predicted for all carotenoids except lycopene. In the testing subset, the correlations were significantly different from one another for α-carotene and β-cryptoxanthin and borderline-significantly different from one another for lutein/zeaxanthin and total carotenoids.

**Table 5:** Spearman correlation coefficients for calculated dietary carotenoid intake ^1^ (*r*1) or predicted plasma carotenoid concentration (*r*2) with measured plasma carotenoid concentration ^2^ in the training (*n* = 2702–2786) and testing subsets (*n =* 1356–1391).

	Training		Testing		
Carotenoid	*r*1	*r*2	*r*1	*r*2	*P* ^3^
α-carotene	0.34	0.41	0.31	0.37	0.0001
β-carotene	0.26	0.30	0.29	0.31	0.35
β-cryptoxanthin	0.34	0.42	0.36	0.41	0.02
Lutein/zeaxanthin	0.26	0.30	0.28	0.31	0.05
Lycopene	0.22	0.21	0.22	0.23	0.81
Total carotenoids	0.20	0.27	0.22	0.27	0.07

^1^ Energy-adjusted, natural log-transformed; ^2^ Natural log-transformed and adjusted for age, case-control status, body mass index, plasma cholesterol, menopausal status, and post-menopausal hormone use by the residual method; ^3^ *P*-value from Wolfe’s test for comparing dependent correlations (*r*1 and *r*2) calculated from probit[rank/(*n* + 1)]-transformed carotenoid measures; *P-*values in training subset ≤0.005 for all carotenoids other than lycopene (*P* = 0.34).

ICCs (95% confidence interval) for calculated dietary carotenoid intake over 2 years in 47,233–48,076 NHS participants were 0.51 (0.50–0.51) for α-carotene, 0.62 (0.61–0.62) for β-carotene, 0.58 (0.57–0.58) for β-cryptoxanthin, 0.64 (0.63–0.64) for lutein/zeaxanthin, 0.43 (0.42–0.44) for lycopene, and 0.55 (0.54–0.55) for total carotenoids. ICCs (95% CI) for the predicted plasma carotenoid concentrations over 2 years in 46,829–47,850 NHS participants were 0.54 (0.53–0.54) for α-carotene, 0.60 (0.59–0.60) for β-carotene, 0.56 (0.55–0.57) for β-cryptoxanthin, 0.62 (0.61–0.62) for lutein/zeaxanthin, 0.43 (0.42–0.44) for lycopene, and 0.59 (0.58–0.59) for total carotenoids. As reported previously, the ICCs for two measured plasma carotenoid concentrations over 1–2 years in 40 NHS participants ranged from 0.73 (α- and β-carotene) to 0.88 (β-cryptoxanthin) [20]. Using these ICCs to correct for medium-term random variation, Spearman correlations for calculated dietary intake and measured plasma concentrations corrected for random within-person variation in the training and testing subsets, respectively, were 0.55 and 0.51 for α-carotene, 0.38 and 0.42 for β-carotene, 0.47 and 0.50 for β-cryptoxanthin, 0.36 and 0.37 for lutein/zeaxanthin, 0.41 and 0.44 for lycopene, and 0.31 and 0.34 for total carotenoids. The Spearman correlations for predicted and measured plasma concentrations corrected for random within-person variation in the training and testing subsets, respectively, were 0.66 and 0.61 for α-carotene, 0.44 and 0.45 for β-carotene, 0.58 and 0.56 for β-cryptoxanthin, 0.42 and 0.43 for lutein/zeaxanthin, 0.40 and 0.44 for lycopene, and 0.39 and 0.39 for total carotenoids.

While some significant interactions with fat intake were observed, prediction was only improved for lutein/zeaxanthin (data not shown). Salad dressing significantly modified the relation between both broccoli and raw carrots and plasma lutein/zeaxanthin in the training subset (*P* *=* 0.02, 0.01, respectively) where the associations decreased with higher salad dressing intake. The testing subset lutein/zeaxanthin predicted-measured plasma concentration Spearman correlation increased significantly (*P* *=* 0.03) to 0.32 with the inclusion of the interaction term. To obtain the most precise regression coefficients possible for the empirical prediction models, we ran stepwise selection for each carotenoid among all participants (Table S2). Tomatoes were selected for α-carotene in all women, but they were not selected in the training subset. Peas or lima beans; yams or sweet potatoes; and kale, mustard, or chard greens were selected for β-carotene in all women but not in the training subset. For lutein/zeaxanthin, kale, mustard, or chard greens were selected in all women but not in the training subset, and corn and tomatoes were selected in the training subset but not in all women. Tomato juice, peas or lima beans, and yams or sweet potatoes were selected for total carotenoids in all women but not in the training subset, and corn and tomatoes were selected in the training subset but not in all women. Most regression coefficients for foods selected in both datasets were similar. Spearman correlations with measured plasma concentrations for the calculated dietary intakes and predicted plasma concentrations, respectively, were 0.33 and 0.40 for α-carotene, 0.27 and 0.31 for β-carotene, 0.35 and 0.42 for β-cryptoxanthin, 0.26 and 0.31 for lutein/zeaxanthin, 0.22 and 0.22 for lycopene, and 0.20 and 0.27 for total carotenoids.

To make the linear regression β-coefficients comparable across foods, 1986 and 1990 average food intakes were converted from servings/day to μg/day of each carotenoid from each food. After this conversion, all foods contributing ≥0.5% to the full cohort’s intake of the carotenoid of interest, excluding margarine for β-carotene and cold breakfast cereal for lutein/zeaxanthin, were included in linear regression models (Table S3). In general, β-coefficients varied widely across foods, and intake of some carotenoids from specific foods, such as α-carotene from bananas and β-cryptoxanthin from apples or pears, were strongly related to plasma concentrations of those carotenoids. Additionally, β-coefficients tended to be larger for β-cryptoxanthin than for the other carotenoids.

## 4. Discussion

The empirical prediction models for plasma carotenoids we developed with FFQ food items included foods expected to be major predictors of the individual carotenoids and some foods likely selected by chance or dietary patterns. The Spearman correlations with measured plasma concentration were modestly yet significantly or borderline-significantly different between calculated diet and predicted levels for all carotenoids except β-carotene and lycopene. Our results suggest calculated intake from published nutrient contents adequately represents bioavailable intake of most carotenoids, but α-carotene, β-cryptoxanthin, and, if taking salad dressing intake into account, lutein/zeaxanthin, may benefit slightly from empirical prediction models.

Major food predictors of each plasma carotenoid were expected. Based on percent of variation in plasma concentrations explained in the training subset, raw carrots were most predictive of plasma α- and β-carotene and total carotenoids. Raw and/or cooked carrots have been identified as α- and β-carotene predictors [10,22] and were top contributors to intake of both carotenoids. Raw carrots may have been more informative than cooked because they were consumed more frequently with greater variation in the training subset (data not shown). Orange juice and oranges were most predictive of plasma β-cryptoxanthin, a logical association given previous selection of orange juice as a β-cryptoxanthin predictor in the NHS [22] and the fact that juice and oranges are assessed well by FFQ (FFQ-diet records *r* = 0.84 for orange/grapefruit juice, 0.74 for oranges) [7]. While not the top contributor to intake, romaine or leaf lettuce may have been most predictive of plasma lutein/zeaxanthin because the frequency of and variation in consumption was generally larger than that of the top contributors (data not shown). Tomato sauce’s predictiveness of plasma lycopene follows lycopene’s enhanced bioavailability in processed versus raw tomatoes [23], the large contribution of tomato sauce to the cohort’s lycopene intake, and studies with similar FFQs [22,24].

Some foods with negligible contributions to carotenoid intake and/or inverse regression coefficients also were selected. Chance may be, in part, responsible given that we tested many foods at a *P* < 0.05 model staying criterion. Cucumbers and/or corn had inverse regression coefficients in the β-cryptoxanthin, lutein/zeaxanthin, and total carotenoids models, though they were not significant in the testing subset. Although they contributed ≤1% to intake, bananas, prunes, raw carrots, and oranges may have been selected for some carotenoids due to correlations among foods. Fruits and vegetables are positively associated with the prudent dietary pattern [25], and in men utilizing a similar FFQ, the prudent dietary pattern was positively correlated with plasma carotenoid concentrations while the Western dietary pattern was inversely correlated [26]. Pizza, which contributed ≤1% to β-carotene intake and had an inverse regression coefficient, is associated with a Western dietary pattern [25] Popcorn contributed <1% to lutein/zeaxanthin intake and is in a food group (snacks) associated with a Western dietary pattern [25] but was significantly positively associated with plasma lutein/zeaxanthin. Popping and likely co-consumption of butter/oil may enhance popcorn’s lutein/zeaxanthin bioavailability, but these hypotheses require further investigation, especially given no observed interaction between fat and popcorn in relation to plasma lutein/zeaxanthin.

The correlations with measured plasma concentrations were only modestly different between predicted plasma carotenoid concentrations and calculated dietary intakes. Relative errors in the empirical weights and nutrient database likely varied across foods and carotenoids and may have contributed to the equivalency in methods. The adequacy of calculated dietary β-carotene intake in representing plasma concentrations confirms a previous analysis [10]. Here, the most significant improvements were observed for α-carotene and β-cryptoxanthin. The α-carotene prediction model may have performed well because one food (carrots) was the main contributor to intake and main predictor of plasma concentrations. The empirical weights may have less error than α-carotene contents assigned to carrots by accounting for bioavailability and assessment validity and by eliminating the nutrient database. Factors such as storage and cultivar can influence carotenoid content of foods [27], and the sample of carrots used to determine the nutrient database α-carotene content may not represent the mix of carrots our participants consumed. Further, the only other food selected for α-carotene was bananas. Compared to carrots, each μg/day increase in banana α-carotene intake was more strongly associated with increased plasma α-carotene concentrations, possibly due to bioavailability. Accounting for bioavailability may explain the better performance of the α-carotene prediction model over calculated dietary intakes. The β-cryptoxanthin prediction model may have benefitted similarly. Oranges and orange juice were the major contributors to intake and strongest predictors of plasma concentrations. Each μg/day increase in β-cryptoxanthin from several foods was associated with a greater increase in plasma β-cryptoxanthin than was a μg/day increase in the other carotenoids from specific foods in relation to plasma concentrations of those carotenoids. An enhanced apparent bioavailability (e.g., greater increases in plasma levels from the same amount of intake) of β-cryptoxanthin and, to a smaller extent, α-carotene versus β-carotene, has been reported previously [28], further supporting a possible benefit of considering bioavailability when assessing exposure for those carotenoids.

Strengths of this analysis include the large sample size, testing the prediction models in a separate group of participants, and use of repeated, extensive FFQs. There are also limitations to this analysis and thus the empirical prediction models. A large proportion of variation in plasma carotenoid concentrations was unexplained, possibly due in part to using a single plasma measurement per woman. However, fairly high ICCs for the plasma carotenoids over a 1–2 year period (0.73–0.88) suggest a single measurement adequately represents longer-term exposure [20]. While we did not account for all factors known to influence plasma carotenoid concentrations, such as genetic variation [29,30,31], this is expected to reduce precision and not introduce bias because the sources of unexplained variation are not likely related to food consumption frequency. In addition, these models were restricted to non-smokers, and as such, may not apply to populations with a large number of smokers. The empirical prediction models also only apply to this cohort because they are mixtures of biological and behavioral associations. To the extent associations differ in other populations, error will occur. If dietary patterns in another population differ, for instance carotenoid-containing foods are more frequently consumed with fats, different conclusions regarding the correlations between biomarkers and predicted *vs.* calculated carotenoid intake may be reached. Although our testing subset was restricted to the NHS blood subcohort, it is likely the models apply to the full cohort and/or FFQs administered at different times. Our ability to use the empirical prediction models on all FFQs administered to the full cohort may offset the reduced precision from unexplained variation in plasma carotenoid concentrations, but accommodating changes in food items on the FFQ over time may introduce additional error. In addition, this approach does not address any inherent measurement error in the FFQ. Finally, these analyses require both measured biomarkers as well as calculated intake of carotenoids, as such, this complex approach may not be easily applied in other settings.

## 5. Conclusions

In summary, we confirmed the utility of determining carotenoid intake from published nutrient contents and further validated the NHS FFQ for carotenoid assessment. This validation of FFQs is beneficial, not only because FFQs are easily measured in large populations but also because translating findings from intakes is more feasible than from plasma levels in terms of generating public health messages. Empirical prediction models may modestly improve bioavailable α-carotene, β-cryptoxanthin, and possibly lutein/zeaxanthin assessment. As the prediction models presented here are specific to the NHS, this approach is worth considering in other cohorts where larger improvements may be observed. Finally, although the improvements using empirical prediction models were modest, it would be of interest to assess if these improvements result in any change in association between intake of carotenoids and chronic disease risk.

## Supplementary Files

Supplementary File 1 Supplementary Information (DOCX, 42 KB)
