# Supplementary material for: Food Predictors of Plasma Carotenoids
Source: Nutrients. 2013 Oct 11;5(10):4051–66. doi: 10.3390/nu5104051 (PMC3820058; doi:10.3390/nu5104051)
Supplement: Supplementary File 1 — Supplementary Information (DOCX, 42 KB) [file nutrients-05-04051-s001.docx]

Supplementary Information

**Table S1.** Candidate food predictors for each carotenoid.

| **Food** | **Portion** | **α-Carot.** | **β-Carot.** | **β-Crypt.** | **Lut./Zea.** | **Lycop.** | **Totcar.** |
| --- | --- | --- | --- | --- | --- | --- | --- |
| Apples or pears, fresh | 1 |  |  | X |  |  |  |
| Bananas | 1 | X |  |  |  |  |  |
| Broccoli | 1/2 cup |  | X |  | X |  | X |
| Brussels sprouts | 1/2 cup |  |  |  | X |  |  |
| Cabbage, coleslaw, or sauerkraut | 1/2 cup |  |  |  | X |  |  |
| Cantaloupe | 1/4 melon |  | X |  |  |  | X |
| Carrots, cooked | 1/2 cup | X | X | X | X |  | X |
| Carrots, raw | 1/2 | X | X | X | X |  | X |
| Cereal, cold breakfast | 1 cup |  |  |  | X |  |  |
| Corn | 1 ear or 1/2 cup frozen or canned |  |  | X | X |  | X |
| Cucumber | 1/4 |  |  | X |  |  |  |
| Eggplant/zucchini/other summer squash | 1/2 cup |  | X |  | X |  | X |
| Eggs | 1 |  |  | X | X |  |  |
| Fruit cocktail or other canned fruit | 1/2 cup |  |  | X |  |  |  |
| Grapefruit | 1/2 |  | X |  |  | X | X |
| Green pepper | 1/2 |  |  |  | X |  |  |
| Hawaiian punch, lemonade,  or other non-carbonated fruit drink | 1 glass, bottle, or can |  |  | X |  |  |  |
| Juice, orange | small glass |  |  | X | X |  | X |
| Juice, tomato | small glass |  | X |  |  | X | X |
| Kale, mustard, or chard greens | 1/2 cup |  | X |  | X |  | X |
| Lettuce, iceberg or head | 1 serving |  | X |  | X |  | X |
| Lettuce, romaine or leaf | 1 serving |  | X |  | X |  | X |
| Margarine | 1 pat |  | X |  |  |  |  |
| Oranges | 1 |  |  | X | X |  | X |
| Peaches, apricots, or plums | 1 or 1/2 cup canned |  | X | X |  |  |  |
| Peas or lima beans | 1/2 cup |  | X |  | X |  | X |
| Pizza | 2 slices |  | X |  |  | X | X |

**Table S1.** *Cont.*

| Popcorn | 1 cup |  |  |  | X |  |  |
| --- | --- | --- | --- | --- | --- | --- | --- |
| Prunes | 5–7 dried or 1/2 cup canned |  | X | X |  |  |  |
| Red chili sauce | 1 Tbsp |  |  |  |  | X |  |
| Sherbet, ice milk, or frozen yogurt | 1/2 cup |  |  | X |  |  |  |
| Soup, home-made with bouillon cubes | 1 cup | X |  |  |  |  |  |
| Soup, home-made without bouillon cubes | 1 cup | X |  |  |  |  |  |
| Soup, ready-made from can, package, or restaurant | 1 cup |  | X |  |  |  |  |
| Spinach, cooked | 1/2 cup |  | X |  | X |  | X |
| Spinach, raw | 1 serving |  | X |  | X |  | X |
| Squash, dark orange/yellow (winter) | 1/2 cup | X | X |  | X |  | X |
| String beans | 1/2 cup |  | X |  | X |  | X |
| Supplemental β-carotene, 1990 | mg |  | X |  |  |  | X |
| Tomato sauce | 1/2 cup |  | X |  | X | X | X |
| Tomatoes | 1 | X | X |  | X | X | X |
| Vegetables, mixed | 1/2 cup | X | X |  | X |  | X |
| Watermelon | 1 slice |  | X | X |  | X | X |
| Yams or sweet potatoes | 1/2 cup |  | X |  |  |  | X |

**Table S2.** β-Coefficients (standard error) for plasma carotenoid ^1^ multivariate linear regression models among all participants
(*n* = 4058–4177).

| **Food ^2^** | **α-Carot. ^3^** | **β-Carot. ^4^** | **β-Crypto. ^5^** | **Lutein/Zeax. ^6^** | **Lycop. ^7^** | **Total Carots. ^8^** |
| --- | --- | --- | --- | --- | --- | --- |
| Apples or pears, fresh |  |  | 0.128 (0.024) |  |  |  |
| Bananas | 0.199 (0.032) |  |  |  |  |  |
| Broccoli |  | 0.311 (0.057) |  | 0.204 (0.037) |  | 0.125 (0.034) |
| Cantaloupe |  | 0.360 (0.078) |  |  |  | 0.133 (0.047) |
| Carrots, cooked | 0.427 (0.071) |  |  |  |  |  |
| Carrots, raw | 0.675 (0.029) | 0.341 (0.032) | 0.102 (0.025) | 0.063 (0.020) |  | 0.167 (0.019) |
| Corn |  |  | −0.180 (0.067) |  |  |  |
| Cucumbers |  |  | −0.058 (0.023) |  |  |  |
| Eggplant/zucchini/other summer squash |  |  |  | 0.161 (0.059) |  |  |
| Eggs |  |  |  | 0.116 (0.028) |  |  |
| Juice, orange |  |  | 0.280 (0.016) | 0.097 (0.013) |  | 0.076 (0.012) |
| Juice, tomato |  |  |  |  | 0.249 (0.055) | 0.093 (0.046) |
| Kale, mustard, or chard greens |  | 0.495 (0.225) |  | 0.308 (0.140) |  |  |
| Lettuce, romaine or leaf |  | 0.164 (0.034) |  | 0.190 (0.021) |  | 0.112 (0.020) |
| Oranges |  |  | 0.446 (0.032) | 0.060 (0.024) |  | 0.121 (0.023) |
| Peaches, apricots, or plums |  |  | 0.256 (0.041) |  |  |  |
| Peas or lima beans |  | −0.225 (0.085) |  |  |  | −0.124 (0.050) |
| Pizza |  | −0.377 (0.148) |  |  | 0.608 (0.107) |  |
| Popcorn |  |  |  | 0.060 (0.018) |  |  |
| Prunes |  | 0.096 (0.047) | 0.117 (0.037) |  |  |  |
| Spinach, cooked |  |  |  | 0.265 (0.085) |  |  |

**Table S2.** *Cont.*

| Spinach, raw |  |  |  | 0.220 (0.076) |  |  |
| --- | --- | --- | --- | --- | --- | --- |
| Supplemental β-carotene, 1990 |  | 0.133 (0.017) |  |  |  |  |
| Tomato sauce |  |  |  |  | 0.602 (0.061) | 0.276 (0.049) |
| Tomatoes | −0.061 (0.031) |  |  |  | 0.121 (0.024) |  |
| Yams or sweet potatoes |  | 0.574 (0.174) |  |  |  | 0.272 (0.102) |

^1^ Plasma carotenoid concentrations were natural log transformed and adjusted for age, case-control status, body mass index, plasma cholesterol, menopausal status, and hormone therapy use by the residual method; ^2^ Foods (servings/day, milligrams/day for supplemental β-carotene) selected by stepwise selection from all foods contributing ≥0.5% to intake of the relevant carotenoid in the full cohort with 0.10 significance level to enter and 0.05 significance level to stay; ^3^ Intercept = 3.99, β (SE) for total energy intake = −0.0000710 (0.0000206), model adjusted *R*^2^ = 0.14;
^4^ Intercept = 5.35, β (SE) for total energy intake = −0.0000670 (0.0000233), model adjusted *R*^2^ = 0.09; ^5^ Intercept = 4.12, β (SE) for total energy intake = −0.0000704 (0.0000181), model adjusted *R*^2^ = 0.15; ^6^ Intercept = 5.03, β (SE) for total energy intake = −0.0000702 (0.0000142), model adjusted *R*^2^ = 0.09; ^7^ Intercept = 5.88, β (SE) for total energy intake = −0.0000608 (0.0000162), model adjusted *R*^2^ = 0.05; ^8^ Intercept = 6.83, β (SE) for total energy intake = −0.0000695 (0.0000142), model adjusted *R*^2^ = 0.07.

**Table S3.** β-Coefficients (standard error) ^1^ for plasma carotenoid ^2^ multivariate linear regression models ^3^ with food intakes coded as amount (μg) of the relevant carotenoid from the given food per day including all foods contributing ≥0.5% to intake of the relevant carotenoid in the full cohort (*n* = 4058–4177).

| **Food** | **α-Carot.** | **β-Carot.** | **β-Crypto.** | **Lutein/Zeax.** | **Lycop.** | **Total Carots.** |
| --- | --- | --- | --- | --- | --- | --- |
| Apples or pears, fresh |  |  | 96.3 (18.2) ^4^ |  |  |  |
| Bananas | 69.5 (11.3) ^4^ |  |  |  |  |  |
| Broccoli |  | 3.06 (0.675) ^4^ |  | 1.64 (0.328) ^4^ |  | 0.535 (0.175) ^4^ |
| Brussels sprouts |  |  |  | −0.0249 (0.801) |  |  |
| Cabbage, coleslaw, or sauerkraut |  |  |  | −2.82 (1.98) |  |  |
| Cantaloupe |  | 1.23 (0.318) ^4^ |  |  |  | 0.448 (0.183) ^4^ |
| Carrots, cooked | 1.27 (0.253) ^4^ | 0.165 (0.129) | −0.422 (3.83) | 1.49 (0.958) |  | 0.0549 (0.0491) |

**Table S3.** *Cont.*

| Carrots, raw | 6.59 (0.285) ^4^ | | 1.65 (0.157) ^4^ | 36.4 (8.87) ^4^ | 9.02 (2.70) ^4^ |  | 0.512 (0.0607) ^4^ |
| --- | --- | --- | --- | --- | --- | --- | --- |
| Corn |  | |  | −17.3 (6.75) ^4^ | −1.37 (0.936) |  | −1.10 (0.716) |
| Cucumber |  | |  | −29.4 (12.0) ^4^ |  |  |  |
| Eggplant/zucchini/other summer squash |  | | 0.651 (1.69) |  | 1.20 (0.604) |  | −0.0756 (0.369) |
| Eggs |  | |  | 74.9 (80.3) | 7.15 (1.71) ^4^ |  |  |
| Fruit cocktail or other canned fruit |  | |  | −8.49 (16.1) |  |  |  |
| Grapefruit |  | | 1.33 (0.783) |  |  | 0.417 (0.262) | 0.194 (0.152) |
| Green pepper |  | |  |  | 0.161 (0.688) |  |  |
| Hawaiian punch, lemonade, or other non-carbonated fruit drink |  | |  | −2.29 (8.52) |  |  |  |
| Juice, orange |  | |  | 16.6 (0.935) ^4^ | 4.54 (0.587) ^4^ |  | 1.77 (0.286) ^4^ |
| Juice, tomato |  | | −1.18 (1.91) |  |  | 0.182 (0.0405) ^4^ | 0.0544 (0.0325) |
| Kale, mustard, or chard greens |  | | 0.623 (0.318) |  | 0.190 (0.0881) ^4^ |  | 0.0655 (0.0583) |
| Lettuce, iceberg or head |  | | −2.06 (2.60) |  | −0.910 (0.872) |  | −0.434 (0.542) |
| Lettuce, romaine or leaf |  | 0.802 (0.187) ^4^ | |  | 1.42 (0.169) ^4^ |  | 0.307 (0.0666) ^4^ |
| Oranges |  |  | | 29.4 (2.11) ^4^ | 3.37 (1.45) ^4^ |  | 2.67 (0.561) ^4^ |
| Peaches, apricots, or plums |  | 3.29 (3.30) | | 39.1 (6.17) ^4^ |  |  |  |
| Peas or lima beans |  | −2.49 (0.916) ^4^ | |  | −0.429 (0.307) |  | −0.323 (0.194) |
| Pizza |  | −7.43 (3.55) ^4^ | |  |  | 0.424 (0.0737) ^4^ | 0.0845 (0.0596) |
| Popcorn |  |  | |  | 5.25 (1.50) ^4^ |  |  |
| Prunes |  | 1.75 (0.898) | | 9.48 (2.98) ^4^ |  |  |  |
| Red chili sauce |  |  | |  |  | −0.717 (0.550) |  |
| Sherbet, ice milk, or frozen yogurt |  |  | | 27.7 (29.6) |  |  |  |

**Table S3.** *Cont.*

| Soup, home-made with bouillon cubes | 3.33 (8.22) |  |  |  |  |  |
| --- | --- | --- | --- | --- | --- | --- |
| Soup, home-made without bouillon cubes | 0.637 (8.07) |  |  |  |  |  |
| Soup, ready-made from can, package, or restaurant |  | −0.321 (1.63) |  |  |  |  |
| Spinach, cooked |  | 0.186 (0.249) |  | 0.230 (0.0856) ^4^ |  | −0.0185 (0.0529) |
| Spinach, raw |  | 0.154 (0.398) |  | 0.310 (0.112) ^4^ |  | 0.119 (0.0744) |
| Squash, dark orange/yellow (winter) | 3.38 (1.78) | −0.114 (0.521) |  | 0.569 (0.630) |  | −0.137 (0.177) |
| String beans |  | 0.273 (3.15) |  | 1.41 (1.15) |  | 0.595 (0.695) |
| Supplemental β-carotene, 1990 |  | 1.32 (0.167) ^4^ |  |  |  | 0.147 (0.0989) |
| Tomato sauce |  | −0.523 (1.56) |  | 4.81 (2.60) | 0.308 (0.0305) ^4^ | 0.126 (0.0246) ^4^ |
| Tomatoes | −5.51 (2.51) ^4^ | −0.123 (0.679) |  | 2.87 (1.52) | 0.365 (0.0768) ^4^ | 0.106 (0.0558) |
| Vegetables, mixed | 1.24 (0.924) | −0.144 (0.488) |  | −0.760 (0.984) |  | −0.195 (0.164) |
| Watermelon |  | −0.936 (1.53) | −3.26 (4.41) |  | 0.112 (0.0676) | −0.0351 (0.0558) |
| Yams or sweet potatoes |  | 1.77 (0.635)^4^ |  |  |  | 0.931 (0.375) ^4^ |

^1^ All β-coefficients and standard errors are multiplied by 10,000; ^2^ Plasma carotenoid concentrations were natural log transformed and adjusted for age, case-control status, body mass index, plasma cholesterol, menopausal status, and hormone therapy use by the residual method; ^3^ Model adjusted *R*^2^ = 0.14 (α-carotene), 0.09 (β-carotene), 0.15 (β-cryptoxanthin),
0.09 (lutein/zeaxanthin), 0.05 (lycopene), 0.08 (total carotenoids); ^4^ *P* < 0.05.
